# Efficient Mode Conversion from a Standard Single-Mode Fiber to a Subwavelength-Diameter Microfiber

**DOI:** 10.3390/nano13233003

**Published:** 2023-11-23

**Authors:** Wanling Wu, Huakang Yu, Chunhua Wang, Zhiyuan Li

**Affiliations:** 1 School of Physics and Optoelectronics, South China University of Technology, Guangzhou 510460, China; 202110187129@mail.scut.edu.cn; 2 School of Electrical Engineering and Intelligentization, Dongguan University of Technology, Dongguan 523808, China

**Keywords:** mode conversion, tapered fiber, direct laser writing method, two-photon polymerization, hybrid photonics system, photonic integration, optical interconnect

## Abstract

Efficient mode conversion is crucial for hybrid photonic systems. We present efficient light transition from a standard single-mode fiber (SMF) to a subwavelength-diameter microfiber via a relatively short tapered fiber. Numerical simulations were performed to design the tapered morphology with high transmittance (approximately 86%) for the fundamental modes. The designed tapered fiber was successfully fabricated on the top of a cleaved SMF tip by the direct laser writing (DLW) method. For the 1550 nm wavelength, the transmittance from the standard SMF to the subwavelength-diameter microfiber was determined to be 77%, accompanied by a change in the effective mode area from 38 μm^2^ to 0.47 μm^2^ within a very short length of 150 μm. Our result demonstrated the versatility of the DLW technique for boosting the mode conversion efficiency of fiber-to-chip devices, enabling various applications in the future.

## 1. Introduction

Efficient mode conversion is essential for optimizing the performance of hybrid optical systems, such as fiber-to-chip coupling [1,2,3,4,5,6], optical trapping [7], sub-wavelength focusing [8,9], and super-resolution optical imaging [10]. In particular, the efficient transmission of light waves between different waveguides with large mode–field mismatch, such as photonic integrated circuits (PICs) and off-chip light sources, has always been a fundamental challenge in photonics. Various approaches have been explored to achieve high-performance mode converters, in terms of light intensity, polarization, or spatial distribution. For instance, mode conversion can be implemented using free-space elements, such as optical lenses, spatial light modulators [11], phase plates [12] and metasurfaces [13,14,15]. Typically, a fiber lens is necessary to transform the guided laser beams of standard optical fiber into micrometer (or subwavelength-scale) spots. However, the intrinsic limitation of fiber lens, especially considering the design freedom on the shape and size of the focused spot, restricted its development from high-profile operation, which is required for many situations. Instead, alternative technical approaches, including all-fiber converters based on long-period grating [16], adiabatic couplers [17,18], angled-facet fiber [19] and tapered fiber [20,21], have been proposed for overcoming the difficulties while being feasible for integration.

Tapered fibers were one of the commonly used elements for mode conversions, which could be readily fabricated by physical heating and drawing methods [22] or the chemical wet etching technique [21]. The diameter of tapered fiber changes adiabatically along the taper length in the range of centimeters, with the corresponding transmittance of the fundamental mode close to unity [22,23,24]. The exceptional transmittance and free-standing features enable various applications, such as strain sensors [25], near-field optical probes [26], fiber-to-chip coupling [24], super-continuum generation [27] and optical trapping [28,29]. However, as for its relatively long length and high aspect ratio, a free-standing tapered fiber is not mechanically stable. Therefore, additional support or stitching in the longitudinal direction is necessary for stabilized coupling and easy handling in diverse situations [20,24].

Recently, direct laser writing (DLW) by two-photon polymerization (TPP) appears to be promising since it allows the flexible fabrication of arbitrary three-dimensional structures with full design freedom and high resolution of hundreds of nanometers [30]. The fabrication feasibility of microstructures on optical fiber tips provides another intriguing solution to overcome the aforementioned drawbacks. For example, an exceptional fiber-to-chip configuration, i.e., photonic wire bonds, was successfully developed for hybrid photonic integration via DLW, which was appealing for reducing the coupling cost [31,32]. However, photonic wire bonds suffered from the complex alignment procedure and difficulty for long-term maintenance. Instead, direct fabrication of a free-standing down-taper with a linear down-taper on optical fiber tips was realized, resulting in 90% fundamental mode transmission for a taper length of 250 µm [33]. As mentioned above, further reduction in the taper length as well as aspect ratio would be crucial for ensuring the mechanical stability of the fabricated microstructures so as to eliminate the requirement of extra support or stitching.

Here, we realized efficient mode conversion from a standard SMF to a subwavelength-diameter microfiber via a relatively short tapered fiber. Mimicking the tapered fiber fabricated using the flame brushing technique, the nonlinear shape of the taper was used to efficiently shorten the total transition length. Numerical simulations were performed to facilitate good mode matching with optimized transition morphology of a relatively short length along with acceptable optical losses. Accordingly, we fabricated the designed taper structure on the fiber tip using the DLW technique. The transition losses and misalignment tolerances of the fabricated structure were both identified experimentally.

## 2. Concept and Numerical Simulations

**Figure 1:**
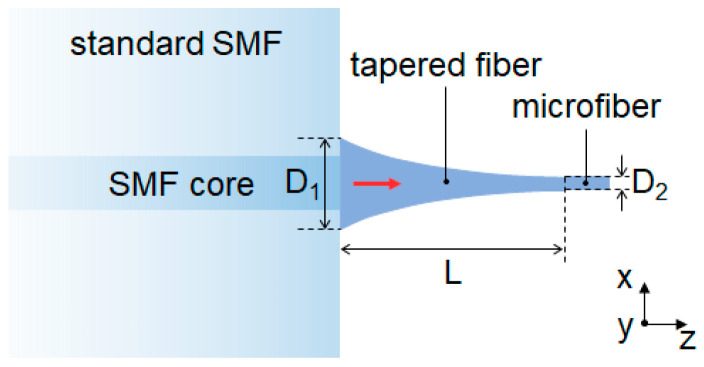
Schematic of the tapered fiber on a cleaved standard SMF tip. A nonlinear tapered fiber was designed to direct the guided light from a standard SMF into a microfiber. The cartesian coordinate is shown with its *z*-direction along the optical axis of optical fiber.

Figure 1 illustrates a schematic diagram of the tapered fiber on a cleaved end facet of a standard single-mode fiber (SMF, 28e+, Corning Optical Fiber Cable Co., Ltd. (Chengdu, China)). The 150 μm length tapered fiber consists of a nonlinearly shaped profile from an input diameter (*D*_1_) of 12 μm to an output diameter (*D*_2_) of 1 (0.5) μm. Mimicking the tapered fiber fabricated using the flame brushing technique, we used an exponential decaying function for defining the nonlinear shape [34].

(1) $$D\left(z\right)=D_{1}e^{-z/L_{0}},$$

where *L*_0_ represents the effective length and *D*_1_ represents the input diameter of the tapered fiber, as shown in Figure 1. The effective length (*L*_0_) is determined by the targeted taper length *L* and input/output diameters (*D*_1_, *D*_2_):

(2) $$L_{0}=-L/\ln\left(\frac{D_{2}}{D_{1}}\right).$$

Such a nonlinear shape is expected to achieve low radiation loss using a relatively short transition length [34]. The end of the taper is connected to a 20 μm length microfiber with a diameter of 1 (0.5) μm for ensuring complete mode conversion to a steadily guided mode. For achieving good mode overlap with the fundamental mode of SMF, the input diameter of the taper section is 12 μm, which is slightly larger than the mode–field diameter (MFD) of the fundamental mode of SMF (~9.5 μm).

**Figure 2:**
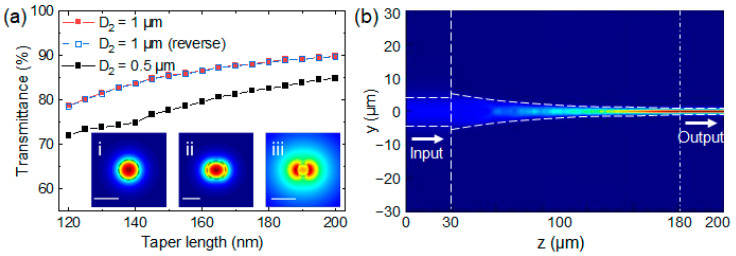
(**a**) Simulated relationship between the taper length and transmittance for input diameter of 12 μm and output diameters of 1 (red squares) and 0.5 μm (black squares) at the wavelength of 1550 nm. The insets present simulated optical field distributions of the fundamental modes with *y*-polarization inside (**i**) SMF and microfibers with diameters of (**ii**) 1 and (**iii**) 0.5 μm at the wavelength of 1550 nm. Scale bar in (**i**) is 10 μm, and scale bars in (**ii**,**iii**) are 1 μm. (**b**) Simulated electric field distribution for a tapered fiber with an output diameter of 1 μm and taper length of 150 μm.

The optical transmittances along with mode evolution were investigated via a Finite-Difference Time-Domain (FDTD) method. The refractive index of the fabricated taper by IP-L resist was set to be 1.5053 [35]. The cross sections of the tapered fiber were assumed to be circular with both output diameters of 1 μm and 0.5 μm investigated. The insets in Figure 2a show the calculated *y*-polarized fundamental modes at a wavelength of 1550 nm for both standard SMF and microfibers with diameters of 1 (0.5) μm. It is noted that the effective mode area of the standard SMF is 38 μm^2^, while the effective mode area of the microfiber is 0.47 (1.41) μm^2^ for a diameter of 1 (0.5) μm.

The influence of taper length (*L*) on transmittance was investigated, as illustrated in Figure 2a. It is clearly seen that a longer taper length would lead to higher light transmittance. This point is reasonable as a longer taper length would render the taper to be adiabatic, which would significantly reduce the radiation loss of guided waves. For the output diameter of 1 μm, 86% transmittance was identified for a relatively short taper length of 150 μm. The corresponding electric field distribution is depicted in Figure 2b. We observed that the mode profile was preserved after passing through the taper section. Additionally, Figure 2a also shows the reverse process, i.e., light transition from the microfiber to the SMF (blue hollow squares). The identical curves indicated the capability of exceptional bidirectional transmission for the designed tapered structure. As shown in Figure 2a, the transmittances for an output diameter of 0.5 μm were also determined, ranging from 72% to 84% for the taper length from 120 μm to 200 μm. These results demonstrate the appealing potential for achieving high coupling efficiency between waveguides with a significant mode–field mismatch.

Considering the wavelength-independent feature of this tapered structure, we investigate the possibility of broadband operation. To demonstrate this point, we have numerically investigated the transmittance for different wavelengths (i.e., 532, 632, 850, 1310 nm) for a fixed taper length of 150 μm and output diameter of 1 μm. Table 1 shows the simulation results, showing the capability of high-transmittance operation over a wide wavelength range. It must be noted that the polymerized resin used in the fabrication is transparent up to 2.4 μm [35], preserving the possibility in the mid-infrared range. Furthermore, the transmittance could be further improved by optimizing the taper length.

**Figure 3:**
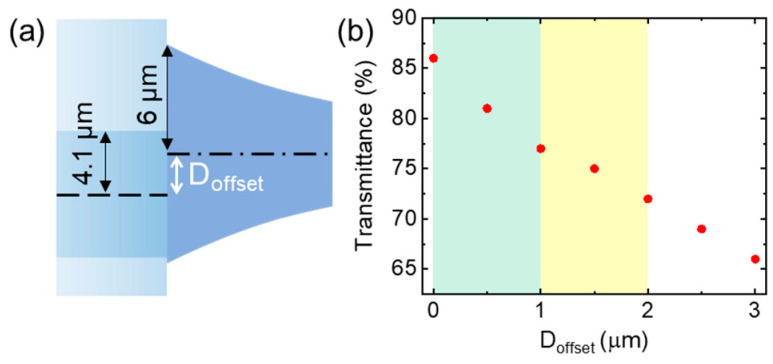
(**a**) Schematic of the lateral offset (denoted with *D*_offset_) between the SMF and taper structure. (**b**) Calculated transmittance as a function of the lateral offset (*D*_offset_).

**Figure A1:**
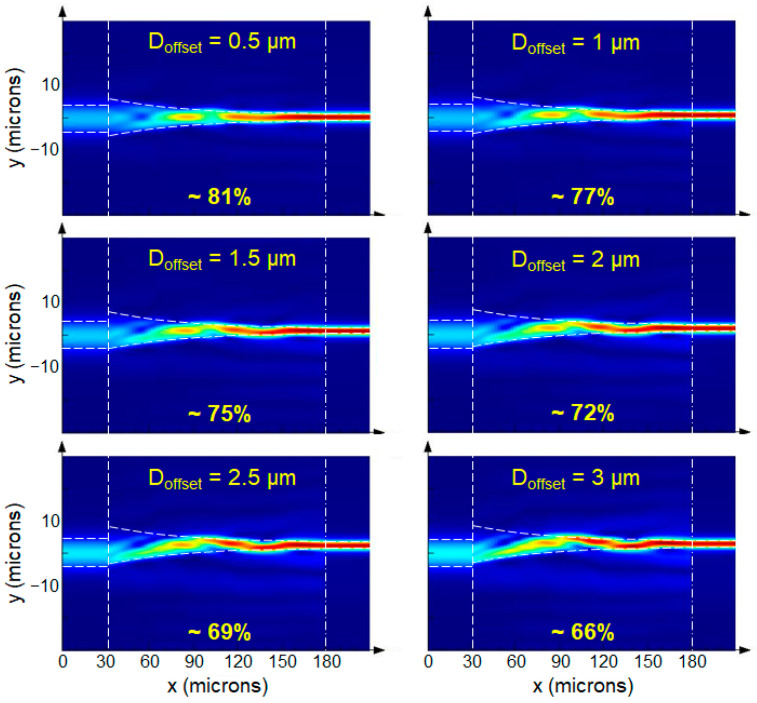
Simulated electric field distributions and corresponding transmittances of designed tapered fibers for different lateral offsets (*D*_offset_) ranging from 0.5 to 3 μm.

Considering practical applications, a misalignment tolerance analysis was carried out in order to investigate the off-axis errors (*D*_offset_ as shown in Figure 3a) between the standard SMF and taper structure due to the fabrication process. Assuming no lateral offset, the simulated transmittance of 86% was obtained. To increase the lateral offset *D*_offset_ as shown in Figure 3b and Figure A1, the simulated transmittance experienced continuous reduction. For the cyan region, a 10% reduction in the transmittance was evaluated for *D*_offset_ up to 1 μm; for the yellow region, a 15% reduction in the transmittance was evaluated for *D*_offset_ up to 2 μm; for the white region, an over 15% reduction in the transmittance was evaluated for *D*_offset_ larger than 2 μm. These findings indicate that the tapered fiber exhibits good tolerance for off-axis alignments, thus reducing the requirement for manufacturing accuracy.

## 3. Experimental Results and Discussion

**Figure 4:**
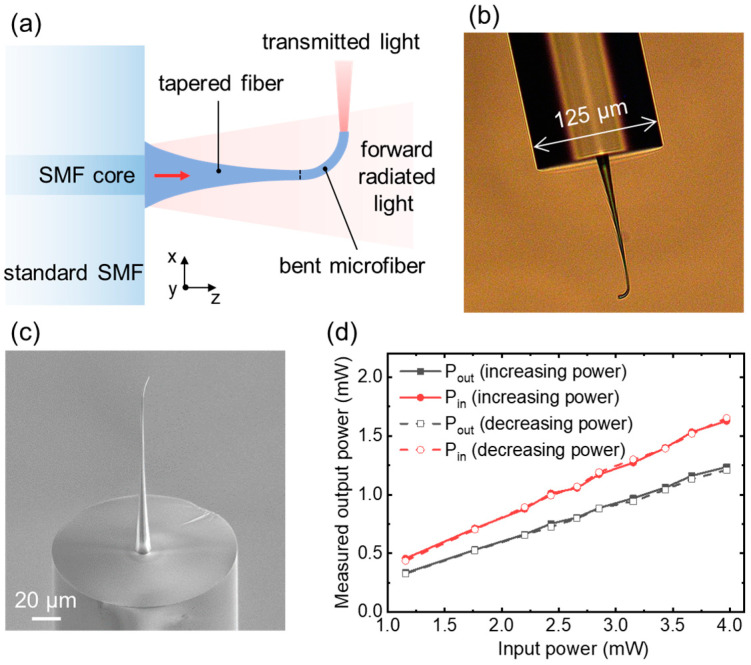
(**a**) Schematic of the fabricated structure. The transition structure included a nonlinear tapered fiber (*L* = 150 μm, *D*_1_ = 12 μm, *D*_2_ = 1 μm) and a circular 90° bent microfiber with a bending radius of 12 μm. (**b**) Microscopic and (**c**) SEM images of the fabricated sample. (**d**) Measured output power as a function of the input laser power for the fabricated sample.

Experimentally, we fabricated the tapered fibers and measured the transmittance accordingly. The tapered fibers were fabricated in an oil immersion configuration by a commercial Nanoscribe Photonic Professional GT system (Nanoscribe GmbH, Eggenstein-Leopoldshafen, Germany). Considering the high aspect ratio of the designed tapered fibers, a slow scan speed (6 mm/s) of the built-in Galvo scanner was chosen in order to reduce the resin’s surface tension and accumulated stress during the printing procedure. The whole structure could be quickly fabricated in 15 min. It must be noted that a bent microfiber, as shown in Figure 4a, was printed and connected to the taper section, which replaces the straight microfiber as shown in Figure 1. From Figure 4a, one can expect the leaked light radiating in the *z*-direction, which would incorrectly increase the measured transmittance if measured along the *z*-direction. With the added bent structure, the guided light would propagate through the circular 90° bent section with a bending radius of 12 μm and emerge from the endfacet in the *x*-direction for measurements. The bent structure would inevitably result in additional bending and insertion losses that should be compensated. Numerically, this amount of additional loss was determined to be 0.086 dB for an output diameter of 1 μm [36]. For determining the transmitted light power, we placed a photodetector (S132C, Thorlabs, Shanghai, China) along the *x*-direction in the far field. Figure 4b,c shows the microscopic and scanning electron microscope (SEM) images of the fabricated structure. These images provide clear visualizations of the transition structure, which included the exponential taper connected by the bent microfiber. It is evident from the images that the surface roughness of the fabricated structures was small, which guaranteed high transmittances.

A 1550 nm wavelength laser from a white light laser (SuperK EXTREME, NKT Photonics, Birkeroed, Denmark) was employed as the light source. During the experiments, transmittances were measured for incident laser power in the range of 1 to 4 mW. To ensure accurate power measurements, the fabricated taper structure together with the standard SMF were mounted on a rotating stage, allowing us to freely adjust the emission direction of guided light from the endfacet. The transmittance, *η*_t_, is determined as

*η*_t_ = *P*_out_/*P*_in_, (3)

where *P*_out_ represents the output power of transmitted light and *P*_in_ represents the power launched into the standard SMF.

The experimental results are shown in Figure 4d. Clearly, *P*_out_ grew linearly with increasing *P*_in_, with the slope corresponding to *η*_t_ ~ 75%. After compensating for the bending losses, the transmittance should be 77% for the pure tapered fiber as shown in Figure 1. The repeatability testing of the device was performed by checking the reversible response. As shown in Figure 4d, we have investigated the transmittance for alternately increasing and decreasing the input power. We observed high consistency for the measurements of increasing and decreasing power, which indicates the good stability and repeatability of the fabricated taper structure. It must be kept in mind that the input power should be limited to a safe range to prevent the damage or accelerated aging of the polymer material.

We tested a number of fabricated tapered fibers, with measured transmittances (after compensating the bending losses) from 67% to 77%. We fabricated six samples for characterizations. The transmittances of three samples were determined to be around 75% with the others around 70%, showing relatively high yields and good repeatability of fabrication. The measured transmittance was lower than the theoretical calculation as shown in the above text, which was possibly attributed to manufacturing errors as a result of misalignment precisions. The same test was performed for all the six samples prepared, all showing excellent reversibility, which represented the good repeatability of our devices. One must keep in mind that the input power should be limited to a safe range to prevent damage of the polymer material. Further improvement of the alignment precision could help minimize off-axis errors, thereby promoting transmittance. In addition, it is important to note that the surface roughness was another contributing factor for the lower transmittance than expected. For the DLW technique, various parameters of the recipes, including femtosecond laser power, scanning speed, and environmental vibrations, would affect the surface roughness of fabricated structures. Surface roughness can be hopefully reduced by further optimizing the recipe, for example, by decreasing the femtosecond laser power or using other optical resists with a high polymerization resolution. Lastly, considering the discrepancy of 15% to 25% compared with the conventional fiber tapers via physical drawing, one may expect that further optimization of the taper morphology, including their longitudinal shape, length, and cross-section, is possible for enhanced optical performance and increased misalignment tolerances.

## 4. Conclusions

Via the DLW technique, we realized efficient mode conversion from a standard SMF to a subwavelength-diameter microfiber with a relatively short taper. Mimicking the tapered fiber fabricated using the flame brushing technique, the nonlinear shape of the taper was used to significantly shorten the total transition length to 150 μm while preserving good transmittance, with a value of ~77%. Numerical simulations were performed to design the tapered morphology with efficient transition efficiency. The misalignment tolerance analysis revealed appreciated transmission tolerance for lateral offset up to 2 μm between the SMF core and taper. The designed tapered fiber was successfully fabricated on the top of the cleaved SMF tip by the DLW method. For the fundamental mode of 1550 nm wavelength, the transmittance from the standard SMF to the subwavelength-diameter microfiber accompanied by a change in the effective mode area from 38 μm^2^ to 0.47 μm^2^ within a very short length of 150 μm. Our result demonstrated the versatility of the DLW technique for boosting the mode conversion efficiency of fiber-to-chip devices, enabling various applications in a more integrated manner. Considering the light transmittance of near unity for conventional fiber taper via physical drawing, further improvement in light transmittance can be expected by optimizing the morphology, improving the alignment precision, and reducing surface roughness.

Benefitting from the fabrication flexibility of DLW technique, the proposed approach can be readily extended to tapered fibers with arbitrary cross sections (e.g., ellipse and rectangle) shapes, not limited to circular cross sections, enabling the establishment of a universal platform for efficient optical transitions with various functions (e.g., polarization manipulation [32]).

## Figures and Tables

**Table 1:** Simulated transmittance for the tapered fiber at different wavelengths.

Wavelength (nm)	Refractive Index of IP-L Resin	Transmittance (%)
532	1.5232	69.83
633	1.5168	80.68
850	1.5105	78.61
1310	1.5062	86.55
1550	1.5053	85.62

Note: The designed taper had the input and output diameters of 12 and 1 μm, respectively, and a length of 150 μm.

## Data Availability

Data underlying the results presented in this paper are not publicly available at this time but may be obtained from the authors upon reasonable request.

## Appendix A. Misalignment Tolerance Analysis

To investigate the misalignment tolerance of off-axis errors, Figure A1 shows simulated electric field distributions and optical transmittances with different lateral offsets from 0.5 to 3 μm. The profile of the tapered fiber was the same as in Figure 2 (*L* = 150 μm, *D*_1_ = 12 μm, *D*_2_ = 1 μm). The results showed that the transmittance could be as high as 66% for *D*_offset_ = 3 μm.
